# Comparison of nutritional status and associated factors of lactating women between lowland and highland communities of District Raya, Alamata, Southern Tigiray, Ethiopia

**DOI:** 10.1186/s40795-017-0179-6

**Published:** 2017-07-17

**Authors:** Ismael Kalayu Sitotaw, Kiday Hailesslasie, Yohannes Adama

**Affiliations:** 1 Department of Public Health, College of Health Sciences, Arsi University, Assela, Ethiopia; 2 Department of Public Health, College of Health Sciences, Mekele University, Mekele, Ethiopia

**Keywords:** Comparison, Anthropometry, Nutritional status, Lactating women, Raya Alamata, Ethiopia

## Abstract

**Background:**

The Ethiopian regions have a relatively higher prevalence of under-nutrition are found in the lowlands of the country, with the exception of the highlands of Tigiray, where under-nutrition is also prevalent.

The intention of this study was to compare anthropometric nutritional status and associated factors of lactating women between lowland and highland communities of district Raya Alamata, Southern Tigiray, Ethiopia.

**Methods:**

A community based comparative cross-sectional study design was conducted from January 27–March 7, 2014. Sample size was determined by two population estimation formula. The total calculated sample size was 456. A stratified sampling technique was used to stratify the study area to highland and lowland. Study participants were selected by simple random sampling technique. Data were collected using anthropometric measurements and structured questionnaire. The raw data were entered and analyzed using SPSS version 20.0. Bivariate and multivariable Logistic regression was done to determine the association between explanatory variable with chronic energy deficiency (CED) using body mass index (BMI), by computing odds ratio at 95% confidence level. A *P* – value <0.05 was considered as statistically significant.

**Result:**

The prevalence of CED of lactating mothers from lowland and highland was 17.5% and 24.6% respectively. After multivariable logistic regression: age, husband occupation, taking vitamin A immediately after delivery or within the first 8 weeks after delivery and consumption of extra food during lactation time were factors associated with chronic energy deficiency for lowland lactating women whereas parity, number of meals per day and household consumption of iodized salt were factors associated with chronic energy deficiency for highland lactating women.

**Conclusion:**

CED in both comparative studies were a serious public health problem. As it is known food security does not mean nutritionally secured, Therefore, the need to develop nutrition intervention such as nutrition security programs to address under-nutrition in the study area is significant, as it was found food secured participants were slightly vulnerable than food insecure. The dietary diversity score of the participants were very low so that encourage the community about nutrition diversification is substantial for adequate nutrient intake.

## Background

Under-nutrition is a serious public health problem linked to a substantial increase in the risk of mortality and morbidity [1]. Malnutrition is one of the most devastating problems worldwide and is inextricably linked with poverty [2]. About 870 million people are estimated to have been undernourished which is 12.5% of the global population. The vast majority of these, 852 million, live in developing countries [3].

Malnutrition is a major public health concern in many developing countries particularly for women responsible for a significant proportion of morbidity and mortality in the affected countries [4]. The nutritional and health status of women is of great concern in the contemporary world, because the multiple roles played by women give rise to serious health and nutritional problems [5].

Women are generally vulnerable to under-nutrition especially during pregnancy and lactation where the food and nutrient requirements increased during that period [6]. Malnutrition passes from one generation to the next because malnourished mothers give birth to malnourished infant [7].

Low BMI, indicative of maternal under-nutrition, has declined somewhat in the past two decades but continues to be prevalent in Asia and Africa [8]. Lactating women from developing countries are considered nutritionally vulnerable groups because this period places a high nutritional demand on the mother especially for those mothers who often start their pregnancy in poor nutritional and health status [9, 10]. Poor maternal nutrition is directly associated with mothers lack of resistance to infection and to maternal ill health during child birth particularly among the poor. Ethiopia is one of the poor countries with the highest levels of lactating mother’s malnutrition in Sub-Saharan Africa [11, 12].

The Ethiopian demographic and health survey (EDHS) working paper, reported that the Ethiopian regions have a relatively higher prevalence of under-nutrition are found in the lowlands of the country, with the exception of the highlands of Tigiray, where under-nutrition is also prevalent [13]. However, there is lack of information in Tigiray as well as in Ethiopia to show under-nutrition in lactating women is prevalent in highland than lowland.

Therefore the current study is conducted to compare the prevalence and associated factors of under-nutrition among lactating women from lowland and highland communities. Finally planners, researchers, programmers as well as policy makers will use it as a source of information for their future nutrition intervention.

## Methods

### Study area and period

The study was conducted in district Raya Alamata. It is one of the districts in the Tigiray Region of Ethiopia. It is located 600 km north of Addis Ababa and about 180 km south of the Tigiray Regional capital Mekelle. Altitude in the area ranges from1178 to 3148 m. 75% of the district is lowland (1500 m above sea level or below) and only 25% is found in intermediate highlands (between 1500 and 3148 m above sea level). Shortage of rainfall is a major constraint of agricultural production in the district. Based on the 2007 national census conducted by the Central Statistical Agency of Ethiopia (CSA), this district had a total population of 85,403, of whom 42,483 were men and 42,920 women. The study period was from January 27–March 7, 2014.

### Study design and sample size determination

The study design was community based comparative cross sectional survey. The Sample size was determined using two population estimation formula, that is

$$ \frac{\left({Z}_{1-\raisebox{1ex}{$\alpha $}\!\left/ \!\raisebox{-1ex}{$2$}\right.\sqrt{2 P\left(1- P\right)}+{Z}_{1-\beta}\ \sqrt{P_1\ \left(1-{P}_1\right)+{P}_2\ \left(1-{P}_2\right)}}\right)2}{\left({P}_2-{P}_1\right)2} $$

were P = (P_1_ + P_2_)/2, pooled estimation of P_1_ and P_2_, Z is the value of standard normal distribution which is 1.96, P_1_ and P_2_ is estimated prevalence of chronic energy deficiency that is 31% and 19.1% in the lowland and highland respectively [14]. Assumptions were 95% confidence level, 5% marginal error, Power (1-β): 80%, Non-response rate: 10%. Therefore, the total calculated sample size was 464.

### Sampling procedure

Stratified sampling technique was used to stratify the study area in to lowland and highland. The district has a total of fifteen lowest administrative levels (Kebeles). Among these lowest administrative levels, ten of them were lowlands and five of them were highlands. Since the purpose of this study is to compare the two groups, thus three lowest administrative levels (kebeles) from highland and lowland were selected. The total calculated sample size 464 was shared equally into two; 232 for the lowland and 232 for the highland participants. The two divided sample sizes were distributed to each selected lowest administrative levels (kebeles) using proportional allocation to size (PAS). Finally participants were selected by simple random sampling technique.

### Data collection procedure and quality assurance

Structured questionnaire was prepared from related literatures, and the questionnaire was translated to local language Tigrigna. It was administered to the participants by health professionals who are fluent speakers of the local language. The dietary diversity of the lactating women was collected using women dietary diversity score (WDDS). It is a simple count of food groups that an individual has consumed over the preceding 24 h. It is calculated by summing the number of food groups consumed by the individual respondent over the 24-h recall period. WDDS uses the following nine food groups: starchy staples, dark green leafy vegetables, vitamin A-rich vegetables and fruits, other fruits and vegetables, organ meat, flesh meat and fish, eggs, dairy, and legumes and nuts [15]. Study participants were asked whether or not they had eaten each food group over the last 24 h. The cut off point for the micronutrient adequacy of women’s diet is consumption of at least five of ten food groups [16]. Household food insecurity of study participants was collected using household food insecurity access scale (HFIAS). It is the measure of the degree of food insecurity in the household in the past 4 weeks (30 days). It consists of two types of related questions. The first question type is called an occurrence question. There are nine occurrence questions that ask whether a specific condition associated with the experience of food insecurity ever occurred during the previous 4 weeks (30 days). Each severity question is followed by a frequency of occurrence question, which asks how often a reported condition occurred during the previous 4 weeks. There are three response options representing a range of frequencies (1 = rarely, 2 = sometimes, 3 = often). HFIAS score is calculated for each household by summing each frequency of occurrence question. The maximum score for a household is 27 (the household response to all nine frequency of occurrence questions was “often”, coded with response code of 3); the minimum score is 0 (the household responded “no” to all occurrence questions, the higher the score, the more food insecurity the household experienced. The lower the score, the less food insecurity a household experienced [17]. Then the households were classified as most food secure scores of 0–11; medium food secure = 12–16; and least food secure = 17 or more [18]. Weights of the lactating women were measured to the nearest 0.1 kg with weight measuring scale (Prestige Model) and heights were measured to the nearest 0.1 cm using a wooden height-measuring board with a sliding head bar. During anthropometric measurement Calibrated equipment and standardized techniques [19] was used to take anthropometric (body) measurements on the lactating women. The measurements were taken with the women wearing light clothing and no shoes to minimize error. Weighing scales were checked before and after each measurement for their accuracy by an object with known weight. Pre-test was carried out 5% of the sample size. During data collection data collectors were strictly follow standard measuring procedure to measure height and weight. Questionnaires were checked for their completeness every day after data collection. For data collectors regular supervisions and follow up were carried by supervisors and principal investigator. The diagnostic criteria for chronic energy deficiency were based on BMI which was calculated as weight in kilograms divided by the square of height in meters (kg/m^2^). It was classified according to WHO classification, BMI < 18.5 kg/m^2^ taken as chronic energy deficiency (CED) and BMI 17.00–18.49 kg/m^2^ mild, 16.00–16.99 kg/m^2^ moderate and less than 16.00 kg/m^2^ severe. Those Participants who had BMI normal (18.5–24.49 kg/m^2^), overweight (24.5–30.0 kg/m^2^) and obese (>30 kg/m^2^) were not considered having chronic energy deficiency.

### Data processing and analysis

The raw data were coded, entered, cleaned and analyzed using SPSS version 20. The 95% confidence level was used in significance analysis. The association between each explanatory variable with dependent variable was examined through bivariate analysis, by computing odds ratio at 95% confidence level. Variables from bivariate analysis were selected and transferred to multivariable logistic regression by using preset *p*-value of <0.25 [20]. To identify factors associated with outcome variables, multiple logistic regressions at 95% confidence level was used. A *p*-value <0.05 was considered as statistically significant. Multi-colinearity effect was checked using variance inflation factor (VIF) and the mean VIF > 5 was used as cutoff point [20]. The final model was then tested for its goodness of fit by Hosmer and Lemeshow *p*-value and a *p*-value >0.05 was best fit. Maternal chronic energy deficiency was measured using maternal BMI which was calculated as weight/height^2^ (kg/m^2^); BMI < 18•5 kg/m^2^ was considered to be underweight, while ≥18.5 kg/m^2^ was considered as normal weight. For the purpose of analysis CED was taken as a dichotomous measure based on body mass index cutoff <18.5 kg/m^2^ and above. Since the interest is in identifying women at risk of underweight, the dependent variables were coded as 1 if the woman was underweight (BMI < 18.5 kg/m^2^) and coded as 0 if not.

## Result

### Socio economic and demographic characteristics of the participants

The total numbers of participants for this study were 456 lactating women, 228 participants were from lowland inhabitants and 228 from highland inhabitants. Overall total response rate for lowland and highland were 98.2%. All of the participants (100%) from lowland were Tigrian ethnic group, but 202 (88.6%) and 26 (11.4%) from highland were ethnic group Tigrian and Amhara respectively. The mean age and income of the study participants were 28.3 ± 6.4, and 1829 ± 1338 (mean + SD) for lowland participants and 28.5 ± 5.8 and 1229 ± 685 (mean ± SD) for highland participants respectively (Table 1).

**Table 1:** Percentage distribution of socio-economic and demographic characteristics of the respondents from lowland and highland communities of district Raya Alamata, Southern Tigiray, Ethiopia, 2014

Variable	Residence	
	Lowland	Highland
	Frequency (%)	Frequency (%)
Age		
< 25	90(39.5)	81(35.5)
25–35	107(46.9)	116(50.9)
> 35	31(13.6)	31(13.6)
Marital status		
Married	203(89.0)	202(88.6)
other^a^	25(11.0)	26(11.4)
Household head		
Husband	203(89.0)	202(88.6)
Wife	25(11.0)	26(11.4)
Family size		
< = 3	47(20.6)	40(17.5)
4–6	119(52.2)	133(58.3)
> = 7	62(27.2)	55(24.1)
Education level of mother		
Illiterate	133(58.3)	167(73.2)
Able to read and write	24(10.5)	21(9.2)
Elementary school (1–6)	23(10.1)	16(7.0)
Junior high school (7–8) & above	48(21.1)	24(10.5)
Education level of husband	*n* = 202	*n* = 203
Illiterate	108(53.5)	96(47.3)
Able to read and write	47(23.3)	52(25.6)
Elementary school (1–6)	15(7.4)	25(12.3)
Junior high school (7–8) & above	32(15.8)	30(14.8)
Occupation of mother		
Housewife only	140(61.4)	64(28.1)
Farmer	73(32.0)	122(53.5)
Other^b^	15(6.6)	42(18.4)
Occupation of husband	*n* = 202	*n* = 203
Farmer	123(60.9)	126(62.1)
Merchant/Trade	31(15.3)	35(17.2)
Private organization employee	25(12.4)	25(12.3)
Other^c^	23(11.4)	17(8.4)
Have TV/Radio/Mobile		
yes	153(67.1)	91(39.9)
No	75(32.9)	137(60.1)

^a^Single, divorced, widowed

^b^Merchant, private organization employee, Government employee, daily labor

^c^Government employee and daily labor

### Agro-ecological characteristics of the participants

About 186 (81.6%) of lactating women from lowland and 192 (84.2%) of lactating women from highland obtained their source of food either, own production or shared production. Regarding own farmland 168 (73.7%) of lactating women from lowland and 166 (72.8%) of the lactating women from highland had their own farmland (Table 2).

**Table 2:** Percentage distribution of agro-ecological characteristics of the respondents from lowland and highland communities of district Raya Alamata, Southern Tigiray, Ethiopia, 2014

Variable	Residence	
	Lowland	Highland
	Frequency (%)	Frequency (%)
Source of food		
Own/shared production	186(81.6)	192(84.2)
Purchase/Market/relative	42(18.4)	36(15.8)
Own farmland		
Yes	168(73.7)	166(72.8)
No	60(26.3)	62(27.2)
Size of farmland	*n* = 168	*n* = 166
< = 0.25	64(38.1)	51(30.7)
0.26–0.75	79(47.0)	103(62.0)
> = 0.75	25(14.9)	12(7.2)
Vegetable production	*n* = 168	*n* = 166
Yes	12(7.1)	17(10.2)
No	156(92.9)	149(89.8)
Grow pulses	*n* = 168	*n* = 166
Yes	14(8.3)	134(80.7)
No	154(91.7)	32(19.3)
Number of cultivation	*n* = 168	*n* = 166
Once a year	131(78.0)	150(90.4)
Two/three times a year	37(22.0)	16(9.6)

### Maternal health and nutrition characteristics

The majority of lactating women 139 (61.0%) from lowland and 128 (56.1%) from highland of the study participants followed antenatal care in the last pregnancy prior to data collection. 118 (51.8%) of lactating women from lowland and 83 (36.4%) of lactating women from highland took vitamin A supplementation immediately after delivery or within the first 8 weeks after delivery. 125 (54.8%) of lactating women from lowland and 135 (59.2%) of the participants from highland did not take any extra food during their lactation time (Table 3).

**Table 3:** Percentage distribution of maternal health and nutrition characteristics of the respondents from lowland and highland communities of, district Raya Alamata, Southern Tigiray, Ethiopia, 2014

Variable	Residence	
	Lowland	Highland
	Frequency (%)	Frequency (%)
ANC		
Yes	139(61.0)	128(56.1)
No	89(39.0)	100(43.9)
PNC		
Yes	122(53.5)	99(43.4)
No	106(46.5)	129(56.6)
Took vitamin A		
Yes	118(51.8)	83(36.4)
No	110(48.2)	145(63.6)
Use family planning		
Yes	123(53.9)	117(51.3)
No	105(46.1)	111(48.7)
Sick/ hospitalized		
Yes	14(6.1)	6(2.6)
No	214(93.9)	222(97.4)
Cause of sickness	*n* = 14	*n* = 6
Malaria	7(50)	1(16.7)
Diarrhea	1(7.1)	5(83.3)
Tuberculosis	2(14.3)	
Other^a^	4(28.6)	
Birth interval	*n* = 180	*n* = 184
< 3 years	46(25.6)	56(30.4)
> = 3 years	134(74.4)	128(69.6)
Place of delivery		
Home	93(40.8)	133(58.3)
Health institution	135(59.2)	95(41.7)
Parity		
< = 2	94(41.2)	82(36.0)
3–6	110(48.2)	126(55.3)
> = 7	24(10.5)	20(8.8)
Frequency of meal in a day		
Three times a day	166(72.8)	152(66.7)
Two times a day	32(14.0)	49(21.5)
> Three times a day	30(13.2)	27(11.8)
Consume extra food?		
Yes	103(45.2)	93(40.8)
No	125(54.8)	135(59.2)
No. of extra food taken	*n* = 103	*n* = 93
One	70(68.0)	52(55.9)
Two/three	33(32.0)	41(44.1)
Type of salt used		
Rock salt	17(7.5)	66(28.9)
Powdered salt	47(20.6)	26(11.4)
Imported iodized salt	23(10.1)	20(8.8)
Local packed iodized salt	141(61.8)	116(50.9)
Use insecticide treated net		
Yes	196(86.0)	44(19.3)
No	32(14.0)	184(80.7)

^a^Cough and fever

### Environmental sanitation and safe drinking water characteristics of the participants

About 161 (70.6%) households of the participant from lowland and 166 (50.9%) of participants from highland had their own latrine. Around 95 (41.7%) and 133 (58.3%) of participants from lowland their sources of drinking water were from tap water and public water respectively and 78 (34.2%), 35 (15.4%), 93 (40.8%) and 22 (9.6%) participants from highland their source of drinking water were public water, protected spring/well, river water and unprotected spring/well respectively. The above explanation and the rest of other environmental sanitation and safe drinking water were shown below (Table 4).

**Table 4:** Percentage distribution of environmental sanitation and safe drinking water characteristics of the respondents from lowland and highland communities of district Raya Alamata, Southern Tigiray, Ethiopia, 2014

Variable	Residence	
	Lowland	Highland
	Frequency (%)	Frequency (%)
Have latrine		
Yes	161(70.6)	116(50.9)
No	67(29.4)	112(49.1)
Type of latrine		
Traditional pit	126(78.3)	97(83.6)
Ventilated improved pit	35(21.7)	19(16.4)
Solid waste disposal		
Open field	53(23.2)	138(60.5)
Dumping	25(11.0)	17(7.5)
In a pit	150(65.8)	42(18.4)
Burning	0	31(13.6)
Liquid waste disposal		
Open field	63(27.6)	145(63.6)
In a pit	165(72.4)	83(36.4)
Main source of drinking water		
Pipe (tap water)	95(41.7)	0
Public water	133(58.3)	78(34.2)
Protected spring/well	0	35(15.4)
River water	0	93(40.8)
Unprotected spring/well/rain	0	22(9.6)
Water storage		
Plastic container/jerican	227(99.6)	201(88.2)
Clay pot	1(0.4)	27(11.8)
Water for cooking, washing		
Pipe (tap water)	95(41.7)	0
Public water	133(58.3)	71(31.1)
Protected spring/well	0	28(12.3)
Unprotected spring/well	0	35(15.4)
River water	0	94(41.2)
Time to fetch water		
0–15 min	76(57.1)	90(39.5)
16–30 min	32(24.1)	62(27.2)
> = 30 min	25(18.8)	76(33.3)

### Household food insecurity of the study participants

Household food insecurity of the participants was measured using household food insecurity access scale. 224 (98.2%) of the study participants from lowland and 225 (98.6%) of participants from highland in the current study were food secure.

### Dietary diversity score of the study participants

Dietary diversity score of the lactating women from the current study is calculated by summing the number of food groups consumed by the individual respondent over the 24-h recall period. Thus 205 (89.9%) of lactating women from lowland and 227 (99.6%) of lactating women from highland had their dietary diversity score less than 5 food groups. The mean dietary diversity score of participants were 1.1 ± 0.302, and 1.00 ± 0.066 for lowland and highland respectively.

### Anthropometric statuses of the study participants

The mean height, weight, and BMI of the study participants were 158.9 cm (±5.8), 51.5 kg (±6.6), and 20.4 kg/m^2^ (±2.4) for lowland communities, and 155.8 cm (±6.1), 49.5 kg (±5.9), and 20.4 kg/m^2^ (±2.1) for highland communities respectively. The prevalence of chronic energy deficiency in lactating mothers from lowland and highland was 17.5% (95% CI (12.7, 22.8) and 24.6% (95% CI (18.6, 29.6) respectively. Even though the magnitude of chronic energy deficiency in both lowland and highland community of the study area was not statically significant the prevalence of underweight in highland lactating mothers was higher than lactating women in the lowland. About 11 (4.9) and 3 (1.3%) of study participants had a BMI greater than or equal to 25 kg/m^2^ for lowland and highland communities respectively (Table 5).

**Table 5:** Percentage distribution of anthropometric status of the study participants from lowland and highland communities of district Raya Alamata, Southern Tigiray, Ethiopia, 2014

	Residence	
	Lowland	Highland
Anthropometric variables	Frequency (%)	Frequency (%)
Maternal height		
≤ 145 cm	1(0.4)	9(3.9)
> 145 cm	227(99.6)	219(96.1)
Maternal weight		
≤ 45 kg	36(15.8)	63(27.6)
> 45 kg	192(84.2)	165(72.4)
Maternal BMI		
< 18.5 kg/m^2^	40(17.5)	56(24.6)
18.5–24.99	177(77.6)	169(74.1)
≥ 25 kg/m^2^	11(4.8)	3(1.3)
Mean	20.4 ± 2.4	20.4 ± 2.1

### Factors associated with chronic energy malnutrition

After multivariable logistic regression, age of the mother, husband occupation, supplementing vitamin A immediately after delivery or within the first 8 weeks after delivery and taking extra meal during lactation were factors associated with chronic energy deficiency of lactating women living in the lowlands. Whereas parity, frequency of meal per day and household consumption of iodized salt used were associated factors of chronic energy deficiency of lactating women living in the highlands of the study area. Thus, from the current study the analysis showed that those lactating women in the age group of 25–35 from lowland were 86.1% at reduced risk to be chronic energy deficient than those lactating women from lowland in the age group of <25, adjusted odds ratio [AOR: 0.139 (0.036, 0.540)]. Those lactating women from lowland who do have partners working in private organization were 89.5% at reduced risk to be chronic energy deficient than those lactating women from lowland who do have partners working in farming [AOR: 0.105 (0.016, .694)]. Those lactating women from lowland who did not took vitamin A immediately after delivery or within the first 8 weeks after delivery were 2.78 times more likely to be chronic energy deficient than those lactating women from lowland who took vitamin A immediately after delivery or within the first 8 weeks after delivery [AOR: 2.78 (1.1, 7.4)]. Those lactating women from lowland who did not took any additional meal during lactation time were 20.64 times more likely to be chronic energy deficient than those lactating women from lowland who took any additional meal during their lactation time [AOR: 20.64 (5.2,81.7)]. Those lactating women from highland who had parity 3–6 were 55% at reduced risk to be chronic energy deficient than those lactating women from highland who had parity less than two [AOR: 0.45 (0.2, 0.9)]. Those lactating women from highland who ate two times a day were 4.4 times more likely to be chronic energy deficient than those lactating women from highland who ate three times a day [AOR: 4.4(1.8, 10.3)]. Those lactating women from highland who used non-iodized salt for their household consumption were 2.45 times more likely to be chronic energy deficient than those lactating women from highland who used iodized salt for their house hold consumption [AOR: 2.45 (1.04, 6.0)] (Table 6).

**Table 6:** Determinant factors of chronic energy deficiency of lactating women from lowland and highland communities of district Raya Alamata, Southern Tigiray, Ethiopia, 2014

Variables	Chronic energy deficiency							
	Lowland				Highland			
	No	Yes	Crude	Adjusted	No	Yes	Crude	Adjusted
	N (%)	N (%)	OR (95% CI)	OR (95% CI)	N (%)	N (%)	OR (95% CI)	OR (95% CI)
Age								
< 25	67(74.4%)	23(25.6%)	1	1	59(72.8%)	22(27.2%)		
25–35	93(86.9%)	14(13.1%)	0.439(0.21, 0.91)*	0.139(0.036, 0.540)**	92(79.3%)	24(20.7%)		
> 35	28(90.3%)	3(9.7%)	0.3(.087, 1.12)*	0.106(0.01, 1.05)	21(67.7%)	10(32.3%)		
Family size								
< = 3	35(74.5%)	12(25.5%)	1	1	26(65.0%)	14(35.0%)		
4–6	101(84.9%)	18(15.1%)	0.52(0.22, 1.18)*	0.41(0.10, 1.66)	104(78.2%)	29(21.8%)		
> = 7	52(83.9%)	10(16.1%)	0.56(0.21, 1.43)*	0.90(0.12, 6.74)	42(76.4%)	13(23.6%)		
Maternal education								
Illiterate	111(83.5%)	22(16.5%)	1	1	125(74.9%)	42(25.1%)		
Read and write	20(83.3%)	4(16.7%)	1.0(0.31, 3.24)	0.89(0.13, 5.77)	18(85.7%)	3(14.3%)		
Elementary school	21(91.3%)	2(8.7%)	0.48(0.105, 2.19)	0.20(0.02, 1.59)	11(68.8%)	5(31.2%)		
Junior and above	36(75.0%)	12(25.0%)	1.68(0.75, 3.73)*	0.39(0.08, 1.94)	18(75.0%)	6(25.0%)		
Husband education								
Illiterate	92(85.2%)	16(14.8%)	1	1	78(81.2%)	18(18.8%)	1	1
Read and write	38(80.9%)	9(19.1%)	1.36(0.55, 3.34)	2.72(0.73, 10.05)	39(75.0%)	13(25.0%)	1.44(0.64, 3.2)	1.19(0.47, 3.01)
Elementary school	12(80.0%)	3(20.0%)	1.43(0.36, 5.66)	1.09(0.13, 9.14)	17(68.0%)	8(32.0%)	2.0(0.76, 5.4)*	1.86(0.62, 5.6)
Junior and above	24(75.0%)	8(25.0%)	1.91(0.73, 5.00)*	1.84(0.42, 8.08)	21(70.0%)	9(30.0%)	1.8(0.73, 4.7)*	2.32(0.71, 7.56)
Husband occupation								
Farmer	100(81.3%)	23(18.7%)	1	1	102(81.0%)	24(19.0%)		
Merchant/trade	24(77.4%)	7(22.6%)	1.26(0.48, 3.29)	1.06(0.26, 4.23)	25(71.4%)	10(28.6%)		
Private org. Employee	23(92.0%)	2(8.0%)	0.37(0.08, 1.71)*	0.105(0.016, .694)**	15(60.0%)	10(40.0%)		
Other	19(82.6%)	4(17.4%)	0.91(0.28, 2.94)	0.32(0.06, 1.51)	13(76.5%)	4(23.5%)		
Have TV/Radio/Mobile								
No	62(82.7%)	13(17.3%)			99(72.3%)	38(27.7%)	1.55(0.8, 2.9)*	1.83(0.79, 4.27)
Yes	126(82.4%)	27(17.6%)			73 (80.2%)	18 (19.8%)	1	1
Supplementing vitamin A								
No	85(77.3%)	25(22.7%)	2.02(1.01, 4.07)*	2.78(1.1, 7.4)**	111(76.6%)	34(23.4%)		
Yes	103(87.3%)	15(12.7%)	1	1	61(73.5%)	22(26.5%)		
Parity								
< = 2	74(78.7%)	20(21.3%)	1	1	58(70.7%)	24(29.3%)	1	1
3–6	95(86.4%)	15(13.6%)	0.58(0.28, 1.21)*	0.67(0.15, 2.86)	102(81.0%)	24(19.0%)	0.56(0.2, 1.0)*	0.45(0.2, 0.9)**
> = 7	19(79.2%)	5(20.8%)	0.97(0.32, 2.93)	2.22(0.19, 24.79)	12(60.0%)	8(40.0%)	1.61(0.58, 4.4)	1.14(0.32, 4.03)
Frequency of meal								
Two times a day	27(84.4%)	5(15.6%)			27(55.1%)	22(44.9%)	3.7(1.87, 7.5)*	4.4(1.8, 10.3)**
Three times a day	136(81.9%)	30(18.1%)			125(82.2%)	27(17.8%)	1	1
> Three times a day	25(83.3%)	5(16.7%)			20(74.1%)	7(25.9%)	1.62(0.62, 4.2)	1.89(0.61, 5.85)
Consume extra meal								
No	90(71.4%)	36(28.6%)	10.01(3.42, 29.2)*	20.64(5.2, 81.7)**	93(68.9%)	42(31.1%)		
Yes	97(96.0%)	4(4.0%)	1	1	79(84.9%)	14(15.1%)		
Type of salt used								
Rock Salt	15(88.2%)	2(11.8%)			43(65.2%)	23(34.8%)	2.1(1.09, 4.2)*	2.45(1.04, 6.0)**
Powdered Salt	37(78.7%)	10(21.3%)			19(73.1%)	7(26.9%)	1.49(0.55, 3.9)	1.41(0.38, 5.20)
Imported iodized salt	17(73.9%)	6(26.1%)			17(85.0%)	3(15.0%)	0.71(0.19, 2.6)	0.80(0.18, 3.48)
Local packed iodized salt	119(84.4%)	22(15.6%)			93(80.2%)	23(19.8%)	1	1
Liquid waste disposal								
Open field	51(81.0%)	12(19.0%)			103(71.0%)	42(29.0%)	2.0(1.02, 3.9)*	1.76(0.72, 4.3)
In a pit	137(83.0%)	28(17.0%)			69(83.1%)	14(16.9%)	1	1
Time taken to fetch water								
0–15 min	60(78.9%)	16(21.1%)			64(71.1%)	26(28.9%)	1	1
16–30 min	25(78.1%)	7(21.9%)			47(75.8%)	15(24.2%)	0.78(0.37, 1.6)	0.55(0.21, 1.41)
> = 30 min	17(68.0%)	8(32.0%)			61(80.3%)	15(19.7%)	0.6(0.29, 1.2)*	0.43(0.18, 1.04)

*P-value <0.25 in the bivariate analysis

**P-value <0.05 in the multivariable analysis

## Discussion

The majority of the participants from lowland and highland were food secured, but their frequency of meal intake was inadequate, or many of the participants were reporting not eating enough meals during the day, the probable reason for this finding may be the social desirability biased for the food security questionnaire. Women of reproductive age particularly lactating women were at a higher risk of insufficient micronutrient intake due to their low dietary diversity, as it was found from descriptive data of the current finding those lactating women from highland had low dietary diversity than lactating women from lowland, their low dietary diversity might be the cause for underweight of the participants since underweight was slightly higher in the highland than lowland, in general the dietary diversity of the majority of the participants from lowland and highland were low, this in turn affects their adequate micronutrient intake, thus the current finding was consistent with other study which noted that, micronutrient intakes for women of reproductive age are far from adequate [21]. The low dietary diversity intake of both comparison groups might be due to less frequency of meal per day, lack of taking extra meals during lactation period, dearth of knowledge regarding fruit and vegetable intake, and limited access to fresh-food markets and stores as well as per capita food consumption of participants, this was consistent with studies conducted to assess socioeconomic determinants of dietary patterns in low- and middle-income countries and a study conducted in Nepal [22, 23]. The mean WDDS of the current study both from lowland and highland were less than the mean WDDS of lactating women in a peri-urban area of Nepal and in rural Cambodia [24, 25], this might be due to the difference in the study population, and it might be also differences in socio-economic status.

Pulse plays a key role in providing nutrition to communities throughout the world. They are widely available, low in fat, and high in protein, minerals, and nutrients in the diet. In adequate intake of pulse indicates a deficit of protein rich. Protein deficiency is the causes of CED. Chronic intake of low amount of nutrient and energy may be the cause of underweight. In the current study the prevalence of underweight was high in the highland, even though cultivation of pulses in the highlands was higher, this may be the reason that the cultivated pulses may sold as income generating rather than household consumption.

Household food insecurity of the participants was measured and most of the participants from lowland and highland were most food secured. Slightly the food security status of the participants from highland was higher than their counterparts. But the prevalence of underweight was higher in the highland than in the lowland. The probable reason for this might be food security status in the highland does not mean that they are nutritionally secured.

The magnitude of chronic energy deficiency of lactating women in the lowlands of the current study was statically significantly lower than a study conducted in Ethiopia [14]. The probable reason for this might be difference in study population and study period. The magnitude of CED of lactating women in the highlands of the current study was found to be similar to the above study. The magnitude of CED of lactating women both in the lowland and highland communities were similar to studies conducted in Nekemte referral hospital and in Samre woreda, South Eastern Tigray, Ethiopia [12, 26]. The prevalence of CED in both comparison groups were lower than a study conducted in lactating women living in relief camps in Sri Lanka [27]. The reason behind may be difference in the living condition of the study population since the study population of the previous study were living in relief camps which make them vulnerable to under-nutrition.

Age of the participant was another factor associated with the nutritional status of lactating women. In the current study lowland inhabitant mothers who were aged from 25 to 35 were found to be 56.1% at reduced risk of CED when compared to those mothers aged <25 years. This finding is consistent with a study conducted in India [28]. Another study in Ethiopia, the highest proportion of malnourished women was observed in the youngest age group, followed by the oldest age group, the lowest rate was found in the age group 20–24 years [29]. The potential reasons for this might be in adolescence and young women nutritional needs increase because of their rapid growth that accompanies puberty and the increased demand for iron that is associated with the onset of menstruation.

Husband occupation was found to be one of associated factor in predicting women for the risk of CED. Those lactating women from lowland who have husband’s occupation working in private organization employee were 89.5% at reduced risk to be CED than those lowland lactating women who do have husband’s occupation working in farming. The probable reason for this could be those lactating women who have partners working in private organization employee may have higher income as compared to those lactating women who had partners working in farming. This finding was consistent with a study from India, partner’s occupation was also the major determinant factor for chronic energy deficiency [30]. Another study in Ethiopia, revealed that women who have agricultural worker partner’s were found to be highly vulnerable to the risk of chronic energy deficiency, low risk of chronic energy deficiency was observed on those who have professional partners [31].

Adequate micronutrient intake by women has important benefits for both women and their children. Maternal diet must provide sufficient energy and nutrients to meet the mother’s usual requirements, as well as the needs of the growing fetus [32]. In the current study taking of vitamin A immediately after delivery or within the first 8 weeks after delivery was found to be a significant factor in reducing chronic energy deficiency. Lowland lactating women who did not took vitamin A immediately after delivery or within the first 8 weeks after delivery were more likely to be undernourished than those lactating women who took vitamin A immediately after delivery or within 8 weeks after delivery. This can be strongly argued that Vitamin A is essential for, maintain mucosal surface of the respirator, gastrointestinal and for growth, reproduction and immunity [33, 34].

According to the Essential Nutrition Action (ENA), taking at least two additional meals per day during lactation is recommended for all lactating women. Good maternal nutrition is important for the health and reproductive performance of women and the health, survival, and development of their children [35]. Accordingly in the current study, lactating women from lowland who did not took any additional meal during lactation time were more likely to be chronic energy deficient as compared to lactating women from lowland who took any additional meal during their lactation time. This was again supported from the cross tabulation that beyond half of study participants from lowland and highland were never took any additional meal during their lactation time. The current finding is consistent with a study conducted in Ethiopia below three fourth’s of the study participants did not take any additional meal during their lactation time [26]. Another study conducted in rural Bangladeshi mothers, the participants were not taking any additional foods during their lactation period, moreover a study conducted in Mayan-mar, none of the women consumed dietary supplements during the lactation period [36, 37]. The probable reason for this might be due to lack of awareness of mothers taking additional meals during lactation and these mothers may not have adequate income to afford two addition meals per day during lactation time.

The risk of malnutrition among women could be affected by the number of children they ever born. In the current study, parity was another factor affecting women’s nutritional status. Those lactating women from highland who had parity 3–6 were 55% at reduced risk to be chronic energy deficient as compared to those highland lactating women who had parity less than two. This is consistent with a study conducted in Ethiopia the result showed women who have never had a child (parity 0) and women with at least five children (parity 5+) were at a higher risk of chronic energy deficiency than other women [13]. However, the current study was in contradict with a study conducted in Samre Woreda, South Eastern Tigray, Ethiopia, showed that nutritional status of study participants had no significant association with number of parity [26].

The quantity of foods consumed by mothers has a direct impact on their health and that of their children. The current finding showed that those highland lactating women who eat two times a day were more likely to be chronic energy deficient than those highland lactating women who eat three times a day. This is consistent with a study done in Bangladesh showed that more than one fifth of women could not afford two meals per day [38] and a study conducted in Erute internally displaced persons CAMP Lira- district which indicated that those women who had less frequent meals per day had a greater chance of being underweight [39]. The possible reason for this trend is the lessen calories from fewer meals taken by these individuals.

Iodine deficiency has adverse effects on all population groups, but women of reproductive ageare often the worst affected. From the result of the current study consumption of household iodized salt was significantly associated with the nutritional status of lactating women from highland. Those lactating mothers from highland who used non-iodized salt for their household consumption were more likely to be chronic energy deficient as compared to those lactating mothers who did not used salt fortified with iodine. Therefore the current finding is consistent with a study conducted in Bangladesh, which showed that almost all women who consume iodized salt shows some sign that the nutritional status of this group of women is better than those who never consumed [40].

## Conclusion

Maternal occupation as farmer may have relationship with the nutritional status of the mother. Farm activity is characterized by heavy workload that can affect women’s nutrition and health status by increased energy use accompanied by inadequate food consumption. A heavy workload for women may lead to a poorer diet which affects women’s health and nutrition. The diet may be poor because there is less time for preparation and cooking. Women involving in agricultural activity skipped lunch because they were working in the fields the whole day. This in turn affects their nutritional status because energy intakes are often below recommended values, as it was found from the study large numbers of participants from highland were farmers and they were also underweight.

Dietary diversity intakes of the participants were below the recommendation that might lead the study subjects to poor nutrient intake which in turn affects their nutritional status. Thus majority of the participants both in the lowland and highland had inadequate nutrient intake, since their dietary diversity was less than 5 food groups.

The prevalence of chronic energy deficiency in both comparative study groups was very high and a serious public health problem. The potential risk factors that affect the nutritional status of the participants from lowland were, age of the lactating women, partners occupation, taking vitamin A immediately after delivery or within the first 8 weeks after delivery, taking any additional meal during lactation time. Whereas number of children ever born (parity), number (frequency) of meals per day, use of iodized salt for household consumption were potential factors that affect nutritional status of study participants in the highland communities.

## Abbreviations

ANC: Antenatal Care
AOR: Adjusted Odds Ratio
BMI: Body Mass Index
CED: Chronic Energy Deficiency
CI: Confidence Interval
EDHS: Ethiopian Demographic and Health Survey
HFIAS: Household Food Insecurity Access Scale
PNC: Postnatal Care
SPSS: Statistical Package for Social Science
VIF: Variance Inflation Factor
WDDS: Women Dietary Diversity Score
WHO: World Health Organization
